# In Colore Veritas? Color effects on the speed and accuracy of true/false responses

**DOI:** 10.1007/s00426-021-01528-z

**Published:** 2021-05-29

**Authors:** Lena Nadarevic, Nikoletta Symeonidou, Alina Kias

**Affiliations:** Department of Psychology, School of Social Sciences, University of Mannheim, 68131 Mannheim, Germany

## Abstract

In addition to their perceptual or aesthetic function, colors often carry conceptual meaning. In quizzes, for instance, true and false answers are typically marked in green and red. In three experiments, we used a Stroop task to investigate automatic green-true associations and red-false associations, respectively. In Experiments 1 and 2, stimuli were true statements (e.g., “tables are furniture”) and false statements (e.g., “bananas are buildings”) that were displayed in different combination of green, red, and gray depending on the experimental condition. In Experiment 3, we used true-related and false-related words shown in green, red, or gray. Participants had to indicate the validity (or semantic meaning) of each statement (or word) as fast and as accurately as possible. We expected that participants would perform best when they had to categorize green stimuli as “true” and red stimuli as “false”. The prediction was only confirmed when green and red stimuli were presented within the same context (i.e., same experimental condition). This finding supports the dimension-specificity hypothesis which states that cross-modal associations (here: associations between color and validity) depend on the context (here: the color-context). Moreover, the observed color-validity effects were stronger when participants had to categorize single words instead of sentences and when they had to provide speeded responses. Taken together, these results suggest that controlled processing counteracts the influence of automatic color associations on true/false responses.

Colors are omnipresent in everyday life and shape the perception of our environment. However, colors do not only have a perceptual or aesthetic function; they also communicate information and can thus influence emotions, cognitions, and behaviors (Elliot and Maier 2007; Elliot et al. 2007). According to Elliot et al.’s *color-in-context theory*, color associations are evolutionary prepared (e.g., a brown fruit means “rotten”) or learned (e.g., a red traffic light means “stop”). Moreover, the theory proposes that colors can have different meanings depending on the context. The color red, for example, is associated with attractiveness in the context of sexual relations (e.g., Elliot and Niesta 2008; Elliot et al. 2010), but associated with failure in achievement contexts (e.g., Elliot et al. 2007; Maier et al. 2008). Elliot et al. (2007) even found that red induces an avoidance tendency in the latter context. Participants who were exposed to the color red on the front page of an IQ test chose easier tasks in the test than those who were exposed to other colors on the front page (e.g., gray or green).

Interestingly, although green is the complement to red on the perceptual level (Choudhury 2015), this does not necessarily hold for the conceptual level. Whereas the majority of studies clearly support a red–failure association in achievement contexts (Elliot and Maier 2007; Maier et al. 2008; but see Mehta and Zhu 2009; Moller et al. 2009), findings are mixed regarding an association between green and success in such contexts (Elliot and Maier 2007; Elliot et al. 2007; Moller et al. 2009). Similarly, although there is strong empirical evidence that red signals danger, the results are less clear with regard to a green–safety association (Pravossoudovitch et al. 2014). Besides such studies that have examined color associations with the concepts failure/success and danger/safety, there is little research on the meaning of the complementary colors red and green in other semantic contexts. Thus, possibly, there might be other contexts in which red and green clearly carry opposite meanings. For example, in a study on memory for truth and falsity, Pantazi et al. (2018) claimed that ”*Green* and *red* are generally associated with concepts of truthfulness versus falsity […]” (p. 179). In the present work, we aimed at testing these proposed color–validity associations.

To our knowledge, this is the first study to empirically investigate the proposed associations between the color green and the semantic attribute *true* as well as between the color red and the semantic attribute *false*. This is surprising, as there are several real-world examples that suggest such color–validity associations. At school, teachers mark false answers in red, in soccer the red card signals false behavior, and in quiz shows the true (vs. false) answer is highlighted in green (vs. red). Moreover, when entering the words “true” and “false” into a Google picture search, a large number of images appear that display the word true in green and the word false in red or that show a green tick mark and a red cross mark. Taken together, these examples speak in favor of green–true associations and red–false associations, respectively. However, it is unclear whether these associations are automatic in nature. By the term automatic we mean that these associations are triggered unintentionally and, once activated, are difficult to suppress (for various features of automaticity, see Moors 2016). In three experiments, we used a Stroop-like paradigm to test whether we would find evidence for automatic green–true and red–false associations.

In the classical Stroop task (Stroop 1935), participants are presented with individual color-words (e.g., green, red, blue, yellow) that either appear in the color they denote (e.g., the word green displayed in green) or in a different color (e.g., the word green displayed in blue). Participants have to indicate the color in which the word appears and to do so as fast and accurately as possible. Because reading is typically much more automatized than color naming, word reading tends to interfere with color-naming whenever word meaning and word color are incongruent, thus resulting in slower response times (RTs) and more errors. In contrast, congruency between word meaning and color can facilitate responding, thus leading to faster RTs and less errors. In a similar vein, a Stroop-like paradigm can be used to measure automatic color-meaning associations (e.g., Goodhew and Kidd 2020; Hong et al. 2020; Lorentz et al. 2016; Moller et al. 2009; Pravossoudovitch et al. 2014; Sherman and Clore 2009). For instance, Pravossoudovitch et al. (2014) asked participants to categorize words as *danger* words (e.g., emergency, threat) or *safety* words (e.g., shelter, home). Importantly, the words were displayed in red, green, and gray. The authors found a significant word type by color interaction on participants’ RTs. For the danger words, participants responded fastest when the words appeared in red, whereas for the safety words they responded fastest when they appeared in green. Interestingly, the red-effect for the danger words was much larger than the green-effect for the safety words, suggesting stronger red–danger associations than green–safety associations.

Following Pravossoudovitch et al. (2014), we used a Stroop task to investigate whether people associate red with the attribute false and green with the attribute true, respectively. In three experiments, participants had to provide true/false responses to stimuli presented in green, red, and gray. The chromatic colors only differed in hue but not chroma. Moreover, all colors were matched on lightness. Keeping lightness constant is important because differences in lightness lead to differences in readability against a given background. This in turn affects RTs and may even result in biased true/false judgments (Reber and Schwarz 1999). Moreover, there is empirical evidence that people associate darkness with negativity and lightness with positivity (Lakens et al. 2012; Meier et al. 2004). These lightness–valence associations would potentially confound the results for the color–validity associations, if the colors differed in lightness.

In a series of Stroop tasks that investigated lightness–valence associations, Meier et al. (2004) varied whether the tasks emphasized accuracy (e.g., by means of accuracy feedback) or speeded responses (e.g., by means of instructions and RT feedback or by means of a response deadline). When accuracy was emphasized, lightness–valence associations showed up in participants’ RTs, whereas when speed was emphasized, the associations showed up in participants’ response accuracies. As we aimed to test whether the predicted color–validity associations were reflected in participants’ RTs and accuracies alike, we implemented a similar procedure. The Stroop task in our experiments consisted of two test blocks. In block 1, participants were instructed to focus on speed and accuracy alike. However, because participants could take up to 5 s to respond, block 1 settings did not prompt participants to respond extremely fast. In contrast, this was the case in block 2, which involved a considerably shorter response deadline. In order to account for individual differences in response speed, this deadline varied across participants depending on their RTs in Stroop block 1.

In addition to manipulating stimulus color, stimulus validity, and response deadlines, we also implemented different color contexts within and between our experiments. This manipulation served to test whether green–true and red–false associations (if present), are inherently stable or depend on the color context. Lakens et al. (2012), for example, could show that black is associated with negativity regardless of the context, whereas white is only associated with positivity in the context of black. The authors found empirical evidence for both types of associations when black and white stimuli appeared within the same experimental task (i.e., were manipulated within participants), but not when they appeared in different contexts (i.e., were manipulated between participants). Similarly, when investigating various color associations by means of the implicit association test (IAT, Greenwald et al. 1998), Schietecat et al. (2018b) observed red–negative associations in the context of green, but not in the context of blue. In the latter context, red was associated not only with aggression, but also with enthusiasm, depending on the targets of the IAT. The authors interpreted this finding as evidence for their *dimension-specificity hypothesis* (Schietecat et al. 2018a, 2018b). This hypothesis states that cross-modal associations (e.g., between color and meaning) depend on the dimension of meaning that is most salient (e.g., evaluation, activity, or potency) in a given context and on the relative conceptual distance of opposing target concepts in this context. Importantly, the dimension-specificity hypothesis predicts that cross-modal associations should only become activated if both target dimensions (e.g., color and meaning) are characterized by clear plus and minus polarities.

In the following experiments, the task of the participants was to categorize stimuli presented in different colors (green, red, or gray) as “true” or “false”. On the conceptual level, true and false form polar opposites on the evaluation dimension. Because green and red should also form a plus and a minus pole on this dimension (see Schietecat et al. 2018b), we expected to find evidence for the predicted green–true and red–false associations if both colors appear within the same context. According to the dimension-specificity hypothesis, however, no color–validity associations should emerge in a color context lacking clear polar opposites. For example, as gray appears to be a neutral color when combined with red and green (e.g., Pravossoudovitch et al. 2014), color–validity associations should not be evident when the color context consists of green and gray stimuli or red and gray stimuli, respectively. In order to test the context-dependency of color–validity associations, Experiment 1 implemented three different color conditions between participants (green–red, green–gray, red–gray). In contrast, Experiments 2 and 3 manipulated all colors (i.e., green, red, and gray) within participants, thus enhancing the complexity of the color context. For all experiments, we will describe how we determined our sample sizes, and we will report all data exclusions (if any), all manipulations, and all measures. The materials and the data of all experiments are publicly available online (Nadarevic et al. 2020).

## Experiment 1

In order to investigate the hypothesized associations between the colors green and red with the attributes true and false, we conducted a Stroop task in which participants had to indicate the validity of short statements. Depending on the experimental condition, the statements appeared either in green and gray (green–gray condition), red and gray (red–gray condition), or green and red (green–red condition). These color-context conditions were manipulated between participants. We predicted that if the proposed green–true and red–false associations are context-*in*dependent, a color by validity interaction should emerge in each of the three conditions. In particular, Stroop performance should be higher when true statements are displayed in green than when they are displayed in red (green–red condition) or gray (green–gray condition). Likewise, Stroop performance should be higher when false statements are displayed in red than when they are displayed in green (green–red condition) or gray (red–gray condition). However, if the assumed color–validity associations require reciprocal activation by the opposite color (as predicted by the dimension-specificity hypothesis), the expected Stroop effects should only appear in the green–red condition. Importantly, Stroop performance was measured by the speed and accuracy of participants’ true/false responses. We expected that in a first Stroop block, which did not require particularly fast responses, the effects should appear primarily in participants’ RTs. In contrast, under speeded conditions, which were implemented in a second Stroop block, the effects should primarily appear in the accuracy data.

## Methods

### Power analysis

We calculated the required sample size for the expected interaction of the within-subject factors color and validity by means of G*Power (Faul et al. 2007). Although G*Power does not have a built-in module to directly calculate power for interactions between repeated-measures factors, this can be accomplished with the program’s *Generic F-test* module by means of an iterative procedure.^1^ The required input parameters for this procedure are α, the degrees of freedom (*df*) for the *F*-test, and the non-centrality parameter λ. For within-subject effects, λ is a function of the sample size *n*, the number of repeated measures *m*, the effect size *f*, and the repeated-measures correlation ρ (see Faul et al. 2007). Because λ and the *df* for the error term depend on the sample size, the power analysis requires to increase *n* in a step-wise fashion until the target power is reached. For Experiment 1, we assumed a medium-sized color by validity interaction effect of *f* = 0.25 and a repeated-measure correlation of ρ = 0.50. The number of repeated measures for the tested 2 $\times$ 2 interaction was *m* = 4. Moreover, we set the type-I error probability to α = 0.05 and our target power to 1-β = 0.95. The power analysis indicated that this target power would be reached with a minimum sample size of *n* = 28 per condition (λ = 14, *df*_effect_ = 1, *df*_error_ = 27), i.e., a sample size of *N* = 84 in total.

### Participants

Eighty-three participants were recruited at the University of Mannheim (61 females, 22 males). Participants had a mean age of *M* = 22.9 (*SD* = 5.9) years. Three participants were non-native German speakers, two of whom reported to have very good German skills, and the third reported to have only intermediate German language skills. Because reading speed was important in the experiment, we decided to exclude the latter participant from all analyses. Moreover, we excluded five participants based on their poor performance in the Ishihara’s color vision test. Thus, the final sample comprised 77 participants (green–gray condition: *n*_*1*_ = 24, red–gray condition: *n*_*2*_ = 26, green–red condition: *n*_*3*_ = 27).^2^

### Materials

Sentences containing exemplar-category assignments of the form “*X* are *Y*” (e.g., “bananas are fruits”, “towers are buildings”) served as stimulus material. We started by creating 20 true target statements based on 20 exemplars and 10 categories. We then created an equal number of false statements by exchanging the categories in pairs between the statements (e.g., “bananas are buildings”, “towers are fruits”). In the same way, we also created 12 statements for practice trials (i.e., 6 true and 6 false ones). The statements were always phrased as affirmatives to keep the material consistent. This is important because research suggests that comprehending affirmative sentences and negated sentences involves different cognitive processes (e.g., Beltrán et al. 2019; Tettamanti et al. 2008). For a complete list of statements, see our materials on the Open Science Framework (OSF; https://osf.io/b8wux/). Colors were selected to differ in hue, but not in lightness or chroma (green: LCh[55.187/82.195/136.016], red: LCh[55.187/82.195/40], gray: LCh[55.187/–/–]).

### Design

Participants were randomly assigned to one of three experimental groups, manipulating the set of colors in which statements could appear to form different color-contexts between participants. All participants accomplished two blocks of the Stroop task that were characterized by different response deadlines. In each block, participants categorized true and false statements that appeared in different colors. Thus, the design was a 3 (color context: green–gray vs. red–gray vs. green–red) $\times$ 2 (block: 1 vs. 2) $\times$ 2 (validity: true vs. false) $\times$ 2 (color: green vs. gray, red vs. gray, green vs. red, depending on the color context) design. All factors except for the color context varied within subjects. Participants’ RTs and their accuracy in the categorization task served as dependent variables.

### Procedure

The experiment consisted of two blocks. In each block, true and false statements were presented on a black computer screen in one of two possible colors, which depended on the experimental group. Participants’ task was to judge a statement’s validity as fast and as accurately as possible by pressing the *d* or *k* key. The mapping of responses (true vs. false) to these keys was counterbalanced across participants. For each trial, a fixation cross appeared for 500 ms in the center of the screen followed by the statement. The statement disappeared as soon as participants provided their response or after 5000 ms. In the latter case the message “too slow” was displayed for 1000 ms. The intertrial interval was also 1000 ms. Participants familiarized themselves with the task in a practice phase consisting of 24 trials (i.e., twelve practice statements presented in each of the two colors of the respective color condition). In the following test block 1, 40 test statements were presented in each of the two colors, resulting in 80 trials in this block. The statements appeared in random order and it was randomly determined in which color a statement appeared first. Upon completion of test block 1, participants had a 30-s break, which was followed by a second block of the Stroop task. Block 2 was identical to block 1, except that it involved an adaptive response deadline to prompt speeded responses. For each participant, the deadline was computed as the 60th percentile of their RT distribution for correct responses in test block 1 (see Rinkenauer et al. 2004, for a similar procedure). Participants had another 24 practice trials to get used to the new deadline before the actual test block started, which again comprised 80 trials in total.

After another 30-s break, participants completed a perceptual fluency test. This test served as a manipulation check to make sure that all colors were equally discriminable on the black screen. Participants’ task was to detect the target letter O within a series of the letter X. Each string consisted of exactly five characters, irrespective of whether the target was present (e.g., XXXOX) or absent (XXXXX). If the target was present, it was displayed as 2nd, 3rd, or 4th character within the string. The overall procedure of the fluency test was similar to Stroop block 1, except that the statements were replaced by strings and participants provided yes/no instead of true/false responses, again by pressing the *d* or *k* key. “Yes” and “true” as well as “no” and “false” always shared the same response key. The fluency test comprised 36 trials in total. Half of the trials consisted of target-present strings and the other half of target-absent strings. The strings appeared equally often in each of the two colors of the respective color-context condition and were presented in random order.

Afterwards, participants saw five plates of Ishihara’s Test for Color Deficiency (Ishihara 2003) that were displayed one after the other on the computer screen. For each color plate, participants’ task was to type in the number displayed. Finally, participants were asked to write down their explicit color associations for truth and falsity, if they had any.

## Results

### Ishihara color vision test

On average, participants identified *M* = 4.2 (*SD* = 1.1) of the five Ishihara plates correctly.^3^ Participants with more than two incorrect responses (*n* = 5) were excluded from analyses (see participant section).

### Perceptual fluency test

We analyzed participants’ mean RTs for correct responses of the perceptual fluency test (96% of the responses) by means of a 2 (target: present vs. absent) by 2 (color: green vs. gray, red vs. gray, green vs. red) ANOVA in each color condition. Importantly, string color did not influence participants’ RTs, *F*s < 1, indicating that the selected colors did not differ in perceptual fluency. RTs were also unaffected by target presence, *F*s ≤ 1.82, *p*s ≥ 0.190, ${\eta }_{p}^{2}$ *s* ≤ 0.07, 90% CIs [0.00, 0.28], [0.00, 0.06], and [0.00, 0.20].^4^ Moreover, there was no significant color by target interaction in any condition, *F*s ≤ 1.62, *p*s ≥ 0.214, ${\eta }_{p}^{2}$ *s* ≤ 0.06, CIs [0.00, 0.20], [0.00, 0.15], and [0.00, 0.25].

### Stroop task

#### Response times

**Fig. 1:**
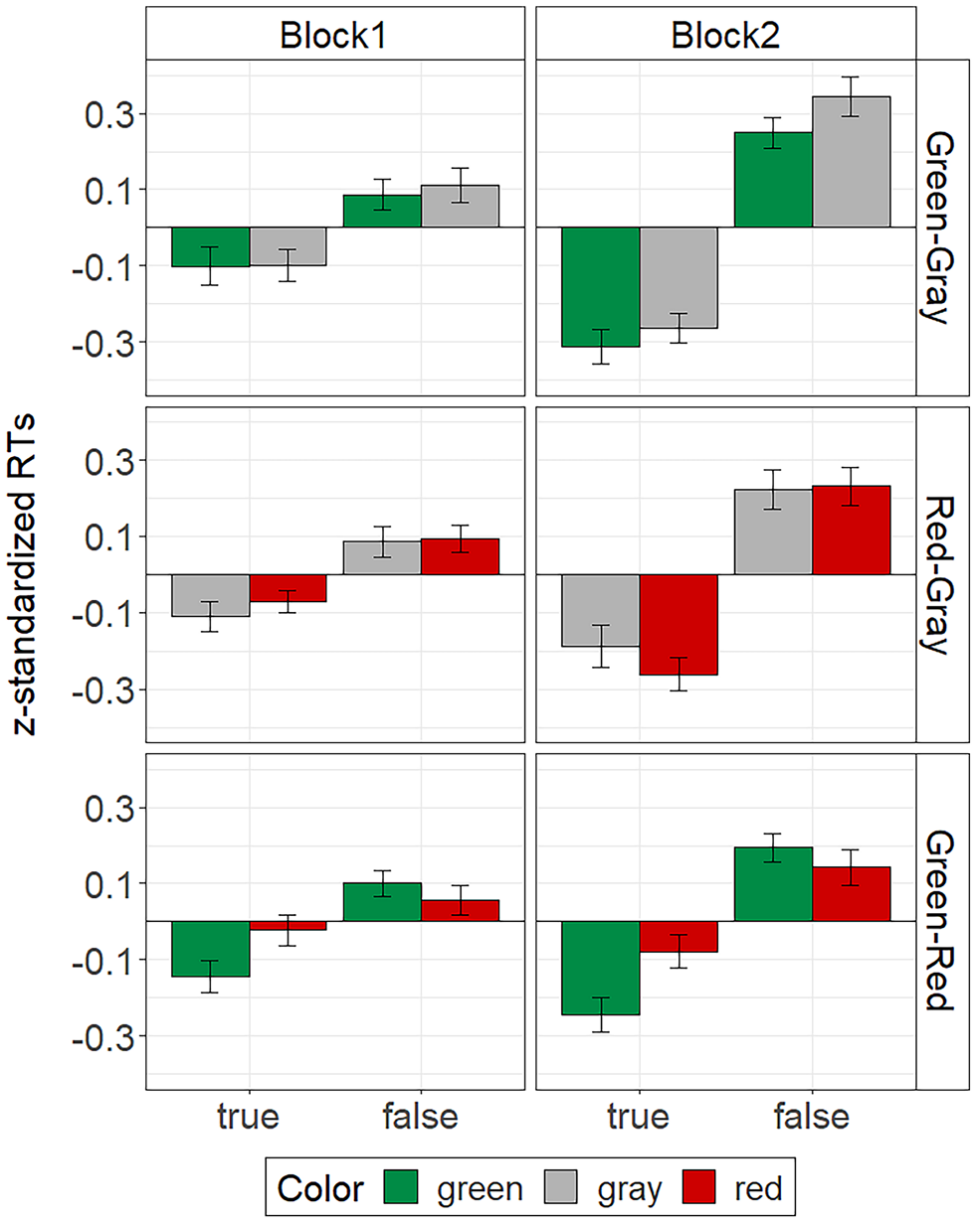
Mean z-standardized RTs in the Stroop task of Experiment 1. The error bars represent standard errors of the means

Before analyzing participants’ RTs in the Stroop task, we excluded all incorrect responses, which were 2.8% of the responses in test block 1 and 15.5% of the responses in test block 2. We then excluded the smallest and the largest RT of each participant in each block to reduce the impact of RT-outliers. According to a simulation study by Bush et al. (1993), this trimming procedure is superior to other outlier exclusion procedures. Due to the different response deadlines in test block 1 (fixed deadline: 5000 ms) and test block 2 (adaptive deadline: *M* = 1048 ms, *SD* = 237 ms) mean RTs and the variability of RTs differed considerably between blocks. To increase the comparability of RT data between blocks, we *z*-standardized RTs in each block as recommended by Bush et al. (1993). That is, RTs were centered around the mean of each participant per block and divided by the participant’s standard deviation in the respective block. We then analyzed these *z*-standardized RTs by means of a 2 $\times$ 2 $\times$ 2 repeated-measures ANOVA with the factors block, color, and validity. We ran the analysis separately for each color-context condition because the different levels of the color factor within each condition (green-gray, red-gray, and green–red) did not allow to include condition as a between-subject factor. The descriptive results are illustrated in Fig. 1. Mean unstandardized RTs per condition are listed in Table 1.

**Table 1:** Mean (SD) unstandardized RTs and error rates for each condition of Experiment 1

Block	Validity	Color	Green-gray condition		Red-gray condition		Green–red condition	
			RTs in ms	Errors in %	RTs in ms	Errors in %	RTs in ms	Errors in %
1	true	green	1041 (294)	4.0 (4.2)	–	–	1046 (285)	4.8 (5.1)
		gray	1025 (265)	2.7 (3.9)	1001 (148)	3.5 (5.1)	–	–
		red	–	–	1010 (156)	4.2 (4.2)	1076 (320)	4.3 (5.8)
	false	green	1081 (291)	1.0 (2.1)	–	–	1092 (254)	1.5 (2.7)
		gray	1078 (295)	1.7 (3.5)	1051 (178)	2.3 (3.5)	–	–
		red	–	–	1059 (192)	1.5 (3.1)	1098 (318)	2.2 (4.0)
2	true	green	727 (129)	13.5 (7.0)	–	–	738 (125)	14.1 (10.0)
		gray	732 (125)	15.2 (10.8)	743 (89)	12.1 (9.6)	–	–
		red	–	–	736 (85)	13.5 (10.0)	758 (149)	18.7 (11.6)
	false	green	786 (153)	17.1 (10.8)	–	–	781 (140)	18.1 (15.5)
		gray	795 (155)	17.9 (10.0)	780 (97)	15.4 (9.3)	–	–
		red	–	–	782 (100)	12.5 (9.6)	777 (146)	17.2 (11.0)

Empty cells were not part of the experimental design

In all color-context conditions RTs were faster for true statements than for false statements, *F*s ≥ 35.83, *p*s < 0.001, ${\eta }_{p}^{2}$ s ≥ 0.59, CIs [0.61, 0.85], [0.36, 0.72], and [0.43, 0.75]. Irrespective of the color context, this validity effect was qualified by test block, *F*s ≥ 6.04, *p*s < 0.021, ${\eta }_{p}^{2}$ s ≥ 0.19, CIs [0.31, 0.70], [0.17, 0.60], and [0.02, 0.40]. Simple main effect analyses showed that the validity effect was stronger in test block 2, *F*s ≥ 35.66, *p*s < 0.001, ${\eta }_{p}^{2}$ s ≥ 0.59, CIs [0.59, 0.84], [0.36, 0.72], and [0.40, 0.74], than in test block 1, *F*s ≥ 11.22, *p*s ≤ 0.002, ${\eta }_{p}^{2}$ s ≥ 0.30, CIs [0.23, 0.66], [0.16, 0.59], and [0.08, 0.50]. There was no color main effect on participants’ RTs, *F*s < 2.50, *p*s ≥ 0.126, ${\eta }_{p}^{2}$ s ≤ 0.09, CIs [0.00, 0.28], [0.00, 0.08], and [0.00, 0.29]. There was also no color by validity interaction effect in the green-gray condition or the red-gray condition, *F*s < 1. In the green–red condition, in contrast, the predicted color by validity interaction emerged, *F*(1, 26) = 9.36, *p* = 0.005, ${\eta }_{p}^{2}$ = 0.26, CI [0.06, 0.47]. Participants in this condition responded significantly faster to true statements displayed in green compared to red (see Fig. 1), *F*(1, 26) = 7.79, *p* = 0.010, ${\eta }_{p}^{2}$ = 0.23, CI [0.04, 0.44]. However, they did not show any RT differences for false statements in green and red, *F*(1, 26) = 2.04, *p* = 0.165, ${\eta }_{p}^{2}$ = 0.07, CI [0.00, 0.27].

#### Accuracy

**Fig. 2:**
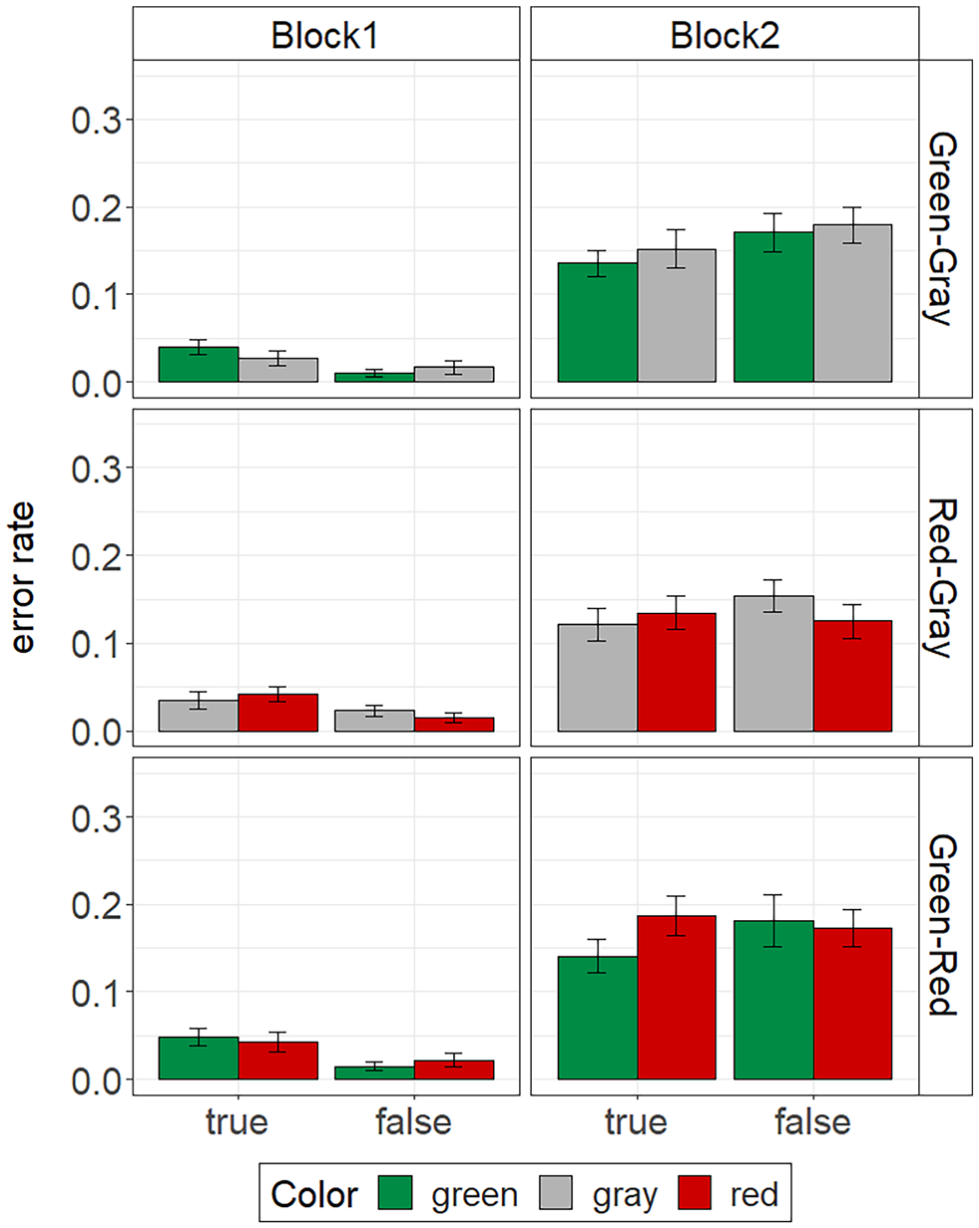
Mean error rates in the Stroop task of Experiment 1. The error bars represent standard errors of the means

In order to examine the effect of the experimental factors block, color, and validity on accuracy, we ran a 2 $\times$ 2 $\times$ 2 repeated-measures ANOVA with mean error rate as the dependent variable. All types of errors (i.e., incorrect responses as well as omission errors) were considered for this analysis. Similar to the RT data, we conducted this ANOVA separately for each color-context condition. The descriptive results are illustrated in Fig. 2.

Not surprisingly, irrespective of the color context, error rates were considerably higher in test block 2 than in test block 1, *F*s ≥ 57.32, *p*s < 0.001, ${\eta }_{p}^{2}$ s ≥ 0.69, CIs [0.66, 0.87], [0.65, 0.86], and [0.50, 0.79]. There was also a block by validity interaction in the green–gray condition, *F*(1, 23) = 7.26, *p* = 0.013, ${\eta }_{p}^{2}$ = 0.24, CI [0.03, 0.46]. The same interaction was evident in the red–gray condition, *F*(1, 25) = 4.13, *p* = 0.053, ${\eta }_{p}^{2}$ = 0.14, CI [0.00, 0.36], and the green–red condition, *F*(1, 26) = 4.23, *p* = 0.050, ${\eta }_{p}^{2}$ = 0.14, CI [0.00, 0.35], but did not reach statistical significance in the two latter conditions. Simple main effect analyses showed that error rates were slightly higher for true statements than for false statements in test block 1, *F*s ≥ 6.56, *p*s ≤ 0.017, ${\eta }_{p}^{2}$ s ≥ 0.21, CIs [0.03, 0.46], [0.02, 0.42], and [0.08, 0.50], but not in test block 2, *F*s ≤ 2.63, *p*s ≥ 0.119, ${\eta }_{p}^{2}$ s ≤ 0.10, CIs [0.00, 0.32], [0.00, 0.18], and [0.00, 0.17]. Similar to the RT data, error rates were unaffected by the color in which a statement appeared, *F*s ≤ 1.25, *p*s ≥ 0.274, ${\eta }_{p}^{2}$ s ≤ 0.05, CIs [0.00, 0.16], [0.00, 0.14], and [0.00, 0.23]. There was also no color by validity interaction effect in any condition, *F*s ≤ 2.93, *p*s ≥ 0.099, ${\eta }_{p}^{2}$ s ≤ 0.10, CIs [0.00, 0.12], [0.00, 0.31], and [0.00, 0.25]. In the green–red condition, however, there was a significant three-way interaction of block, color, and validity, *F*(1, 26) = 5.53, *p* = 0.027, ${\eta }_{p}^{2}$ = 0.18, CI [0.01, 0.39], which did not appear in the other conditions, *F*s < 1. Separate analyses for each test block in the green–red condition revealed a significant color by validity interaction in test block 2, *F*(1, 26) = 4.36, *p* = 0.047, ${\eta }_{p}^{2}$ = 0.14, CI [0.00, 0.35], but not in block 1, *F* < 1. The interaction in block 2 emerged because participants made less errors when categorizing true statements displayed in green compared to red (see Fig. 2, for exact values of mean percent errors per condition see Table 1), *F*(1, 26) = 6.70, *p* = 0.016, ${\eta }_{p}^{2}$ = 0.20, CI [0.02, 0.42], but showed no differences for false statements in green and red, *F* < 1.

### Explicit color–validity associations

Two participants failed to fill out a questionnaire about explicit color–validity associations. Of the remaining 75 participants, 60% indicated that they associated green with truth and even more (76%) indicated that they associated red with falsity, when asked about their color–validity associations in the questionnaire. A complete list of participants’ explicit color associations is displayed in Appendix A.

## Discussion

The results of Experiment 1 suggest that automatic green–true and red–false associations are highly context dependent. We only found empirical evidence for the hypothesized color–validity interaction in the green–red condition. That is, statement color only influenced participants’ RTs in the Stroop task when green and red statements were presented in the same experimental context. For the accuracy data, this interaction additionally depended on test block. Specifically, a color by validity interaction on participants’ error rates in the green–red condition was only evident in block 2, in which participants had to respond very fast. Hence, under speeded conditions, the Stroop effect was evident in the RT data as well as the accuracy data. The observed Stroop effect was characterized by faster responses and less errors for true statements in green compared to red. In contrast, no Stroop effect was evident for the false statements. At first glance, this pattern speaks in favor of a green–true association, but not a red–false association. More general color effects irrespective of validity (e.g., simple green-go and red-stop associations), on the other hand, can be ruled out as an explanation. If participants had associated green with “go” and red with “stop” in the context of the experiment, we should have observed a color main effect on RTs, both in the Stroop task and in the perceptual fluency task. However, neither was the case. In contrast, other interpretations of the data seem plausible, which we outline below.

Overall, participants responded significantly faster to true than to false statements. Although we had not predicted this validity effect, it converges with findings of classical semantic-memory studies (e.g., Collins and Quillian 1969). Furthermore, there is reason to believe that statement verification and falsification rely on qualitatively different processes. For example, Marques et al. (2009) observed different patterns of brain activation during sentence verification and falsification. Verification corresponded with activation in brain regions presumed to be involved in search processes and matching processes with stored information, whereas falsification corresponded with activation in brain regions that are engaged in reasoning processes. Possibly, these more elaborate reasoning processes not only accounted for the slower responses to false statements, but also counteracted the effect of automatic color–validity associations for such statements. This might explain why we only found a color effect for true statements, but not for false statements in the green–red condition. What is more, prior research suggests that the contribution of interference effects to the Stroop effect is typically much stronger than the contribution of facilitation effects (Chen and Johnson 1991). Hence, possibly, the Stroop effect for the true statements reflects an interference effect of the color red rather than a facilitation effect of the color green. Because the two effects can only be separated by means of a reference color, we added gray as the reference color in the following experiments.

## Experiment 2

Experiment 2 was similar to Experiment 1, but this time all participants were presented with statements displayed in green, red, and gray within the same context (i.e., within the same experimental condition). We implemented this change for the following reasons. First, we wanted to test whether the findings of the green–red condition would also replicate in the presence of gray. Considering the assumptions of the dimension-specificity hypothesis, this is by no means trivial. For example, Schietecat et al. (2018b) reasoned that a task with three instead of two colors “reduces the presence of a clear bipolar opposition, and, therefore, the strength of crossmodal associations in such a task might be much smaller, or maybe even zero” (p. 8). Second, in case of a successful replication, gray would serve as a reference condition that would help us to assess the relative contributions of the presumed green-true and red–false associations to the Stroop effect.

## Methods

### Power analysis

We again conducted a power-analysis with G*Power. However, given the findings of Experiment 1, we were now more conservative with regard to the expected effect size of the color by validity interaction (*f* = 0.20). The number of repeated measures for the tested 3 $\times$ 2 interaction was *m* = 6. All other parameters were the same as in Experiment 1 (ρ = 0.50, 1-β = 0.95, α = 0.05). This power analysis suggested a minimum sample size of *N* = 34 participants (λ = 16.3, *df*_effect_ = 2, *df*_error_ = 66).

### Participants

Forty-three participants were recruited at the University of Mannheim (30 females, 13 males). Participants had a mean age of *M* = 23.7 (*SD* = 5.3) years. Five participants were non-native German speakers but reported to have very good German language skills. Moreover, all participants indicated to have full color vision.

### Materials

We selected 12 true statements and 12 false statements from Experiment 1 (i.e., 24 statements in total) as test stimuli. Eight additional statements (four true and four false ones) served as practice stimuli.

### Design and procedure

The experimental design was a 2 (block: 1 vs. 2) $\times$ 3 (color: green vs. red vs. gray) $\times$ 2 (validity: true vs. false) within-subject design. As in Experiment 1, participants’ RTs and their accuracy served as the dependent variables. The procedure was the same as in Experiment 1, except for the following changes: Each of the eight practice statements (four true and four false ones) was displayed in each color (red, green and gray), thus resulting in 24 trials per practice block. Likewise, each of the 24 test statements (12 true and 12 false ones) appeared in each color, thus resulting in 72 trials per test block. The perceptual fluency test involved 36 trials in total with the color of each string and the string type (target present vs. absent) randomly determined for each trial. Finally, this time, the computer presented 15 Ishihara color plates to participants.

## Results

### Ishihara color vision test

On average, participants identified *M* = 13.2 (*SD* = 1.4) of the 15 Ishihara plates correctly. Because all participants indicated to have full color vision and there was no participant with a notably low performance in the Ishihara test, we did not exclude any participants.

### Perceptual fluency test

We analyzed mean RTs for correct responses of the perceptual fluency test (95% of the responses) by means of a 2 (target: present vs. absent) $\times$ 3 (color: green vs. red vs. gray) repeated-measures ANOVA. Importantly, as in Experiment 1, string color did not influence participants’ RTs, *F* < 1. This time RTs were significantly faster for target-absent trials (*M* = 462, *SD* = 96) compared to target-present trials (*M* = 486, *SD* = 108), *F*(1, 42) = 5.30, *p* = 0.026, ${\eta }_{p}^{2}$ = 0.11, CI [0.01, 0.27], but this target effect was not moderated by color, *F* < 1.

### Stroop task

#### Response times

**Fig. 3:**
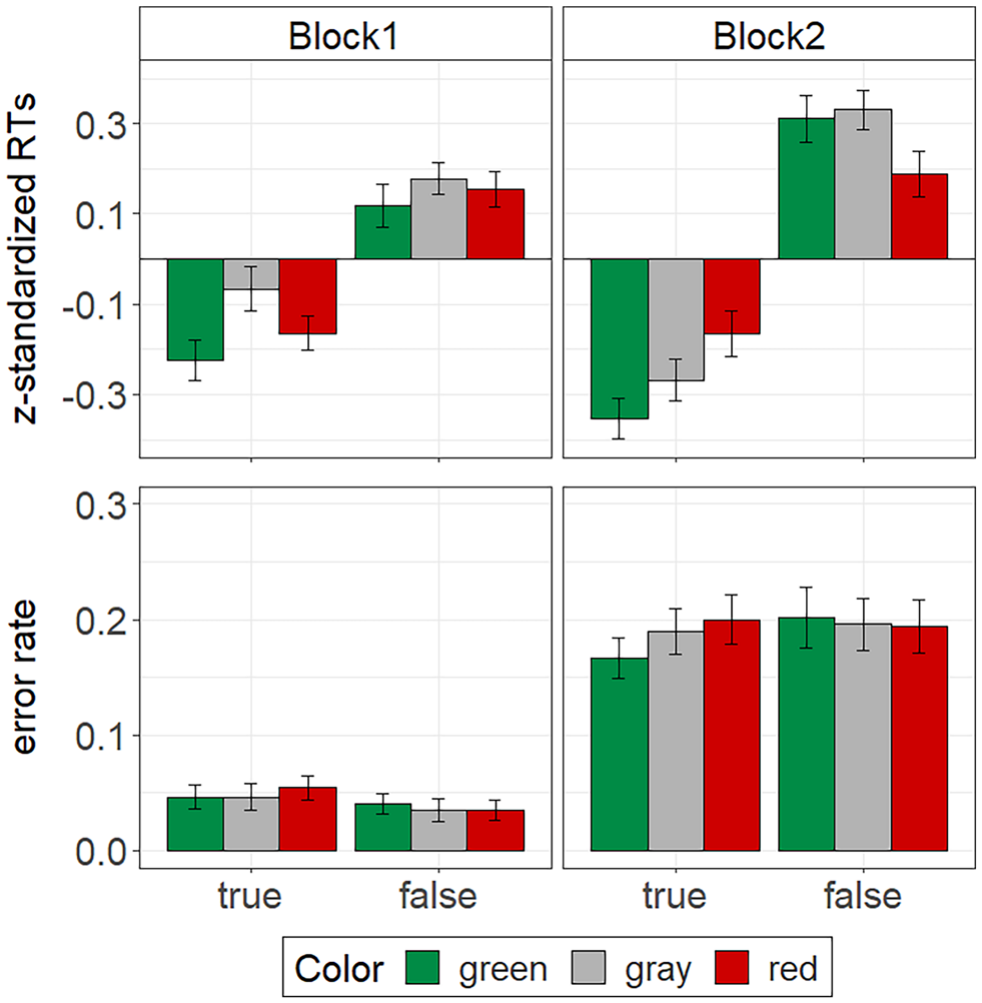
Stroop performance in Experiment 2 as measured by participants’ mean *z*-standardized RTs and participants’ error rates. The error bars represent standard errors of the means

As in Experiment 1, we excluded RTs for incorrect responses (block 1: 4.3%, block 2: 19.1%). We then trimmed RTs by omitting participants’ fastest and slowest response per block. Due to the different response deadlines in test block 1 (fixed deadline: 5000 ms) and test block 2 (adaptive deadline: *M* = 939 ms, *SD* = 155 ms) we again *z*-standardized RTs per block. These *z*-standardized RTs were then submitted to a 2 (block: 1 vs. 2) $\times$ 3 (color: green vs. red vs. gray) $\times$ 2 (validity: true vs. false) repeated-measures ANOVA. We report Greenhouse–Geisser corrected degrees of freedom whenever the sphericity assumption does not hold (as indicated by Mauchly’s test). The descriptive results are displayed in Fig. 3. Mean unstandardized RTs per condition are listed in Table 2.

**Table 2:** Mean (SD) unstandardized RTs and error rates for each condition of Experiment 2

Block	Validity	Color	RTs in ms	Errors in %
1	True	Green	890 (168)	4.7 (6.6)
		Gray	922 (179)	4.7 (7.3)
		Red	895 (153)	5.4 (6.5)
	False	Green	955 (169)	4.1 (5.6)
		Gray	979 (186)	3.5 (6.6)
		Red	975 (195)	3.5 (5.8)
2	True	Green	655 (88)	16.7 (11.6)
		Gray	661 (88)	19.0 (12.9)
		Red	671 (91)	20.0 (14.0)
	False	Green	713 (101)	20.2 (17.4)
		Gray	717 (102)	19.6 (14.5)
		Red	704 (97)	19.4 (14.9)

Similar to Experiment 1, responses were significantly faster for true statements compared to false statements, *F*(1, 42) = 98.22, *p* < 0.001, ${\eta }_{p}^{2}$ = 0.70, CI [0.57, 0.78]. This validity effect was again moderated by test block, *F*(1, 42) = 20.36, *p* < 0.001, ${\eta }_{p}^{2}$ = 0.33, CI [0.14, 0.49]. Simple main effect analyses revealed a large validity effect in block 1, *F*(1, 42) = 44.69, *p* < 0.001, ${\eta }_{p}^{2}$ = 0.52, CI [0.34, 0.64], and an even larger effect in block 2, *F*(1, 42) = 98.49, *p* < 0.001, ${\eta }_{p}^{2}$ = 0.70, CI [0.57, 0.78]. Unlike in Experiment 1, we observed a significant main effect of color, *F*(1.83, 76.80) = 3.56, *p* = 0.037, ${\eta }_{p}^{2}$ = 0.08, CI [0.00, 0.17]. This color effect was qualified by a three-way interaction of color, block and validity, *F*(1.82, 76.53) = 4.09, *p* = 0.024, ${\eta }_{p}^{2}$ = 0.09, CI [0.01, 0.19]. When analyzing the data separately per test block, the color effect did not replicate in either block, *F*s ≤ 2.89, *p*s ≥ 0.067, ${\eta }_{p}^{2}$ s ≤ 0.06, CI [0.00, 0.15] and [0.00, 0.07]. We also did not find a color by validity interaction in block 1, *F* < 1. However, the predicted color by validity interaction appeared in test block 2, *F*(1.97, 82.69) = 5.56, *p* = 0.006, ${\eta }_{p}^{2}$ = 0.12, CI [0.02, 0.22].

To explore the nature of the color by validity interaction in block 2, we conducted simple main effect analyses. Similar to the results of Experiment 1, these analyses indicated no color effect on RTs for false statements, *F*(1.70, 71.39) = 2.66, *p* = 0.085, ${\eta }_{p}^{2}$ = 0.06, CI [0.00, 0.15], but a significant color effect for true statements, *F*(1.95, 81.79) = 3.70, *p* = 0.030, ${\eta }_{p}^{2}$ = 0.08, CI [0.00, 0.18]. Although the descriptive pattern of this color effect for the true statements in block 2 was in line with our expectation (see Fig. 3), planned contrasts to the color gray as the reference condition were not significant. That is, compared to true, gray statements, RTs were neither significantly faster for true, green statements, *F*(1, 42) = 1.47, *p* = 0.233, ${\eta }_{p}^{2}$ = 0.03, CI [0.00, 0.16], nor significantly slower for true, red statements, *F*(1, 42) = 1.96, *p* = 0.169, ${\eta }_{p}^{2}$ = 0.04, CI [0.00, 0.18].

#### Accuracy

We also compared participants’ mean error rate for the different combinations of block, color, and validity by means of a 2 $\times$ 3 $\times$ 2 repeated-measures ANOVA. Not surprisingly, participants made more errors in test block 2 than in test block 1, *F*(1, 42) = 144.36, *p* < 0.001, ${\eta }_{p}^{2}$ = 0.77, CI [0.67, 0.84]. Unlike in Experiment 1, however, there were no other main effects or interactions, *F*s ≤ 1.71, *p*s ≥ 0.198, ${\eta }_{p}^{2}$ s ≤ 0.04. Yet at least descriptively error rates were lower when true statements appeared in green than when they appeared in red (see Fig. 3, for exact values of mean percent errors per condition see Table 2).

### Explicit color–validity associations

When asked about their explicit color–validity associations, 93% of the 43 participants indicated that they associated green with the attribute true. Likewise, 93% of the participants indicated that they associated red with the attribute false. A complete list of participants’ explicit color associations is provided in Appendix A.

## Discussion

Although this time the colors green, red, and gray had been presented in the same experimental context, the pattern of results for the RT data was similar to the one in the green–red condition of Experiment 1. This time, however, the Stroop effect for the RT data was moderated by test block. We did not find evidence for color-validity associations in block 1 of the Stroop task. In contrast, block 2, which required speeded responses, revealed a significant color–validity interaction on participants’ RTs. In line with the results of the green–red condition of Experiment 1, we only found a significant color effect on RTs for true statements, but not on RTs for false statements. Descriptively, RTs for true statements slowed down when these statements appeared in red and speeded up when they appeared in green. However, in both cases, contrasts to the gray baseline condition failed to reach statistical significance. We also did not find significant color-validity interactions on participants’ error rates. Note, however, that the descriptive pattern of the error rates in block 2 also mirrored the one found in Experiment 1. Apparently, in line with the presumptions of Schietecat et al. (2018b), the inclusion of a neutral baseline condition in Experiment 2 had attenuated the color–validity effect. Considering this, the aim of our third experiment was to use material that would likely produce stronger color-validity effects.

## Experiment 3

Experiment 3 aimed to replicate Experiment 2 with a different set of materials. In the style of other studies that investigated color associations with a Stroop tasks (e.g., Goodhew & Kidd, 2020; Hong et al. 2020; Lorentz et al. 2016; Moller et al. 2009; Pravossoudovitch et al. 2014; Sherman & Clore, 2009), Experiment 3 applied words instead of statements as stimuli. The presented words were close semantic associates of the attributes true and false. In line with Experiment 2, these true-related words and false-related words appeared in green, red, and gray and had to be categorized based on their meaning (true vs. false). Because assessing word meaning should require less controlled processing than the evaluation of complete sentences does, we expected Experiment 3 to reflect automatic color–validity associations more clearly than the previous experiments did.

## Method

### Power analysis

Because the experimental design was the same as in Experiment 2, power calculations did not change. Thus, the minimum sample size was set to *N* = 34.

### Participants

Forty-eight participants (37 females, 11 males) were recruited at the University of Mannheim. Participants had a mean age of *M* = 23.6 years (*SD* = 4.8) and were German native-speakers except for one participant. This participant indicated to have very good German skills but showed severe difficulties in understanding the instructions and was thus excluded from all analyses. Additionally, we excluded four participants who showed signs of color-deficiency in the Ishihara test. Hence, the final sample comprised *N* = 43 participants.

### Materials

Twenty true-related words and 20 false-related words were submitted to a pretest (*N* = 23). The pre-testers rated each word according to whether it carries the same meaning as the attributes true or false, respectively (1 = *I strongly disagree*; 7 = *I strongly agree*). In addition, pre-testers rated the familiarity of each word (1 = *not at all*; 5 = *completely*). Considering a comparable word length (*M* = 8.67, *SD* = 1.37) and familiarity (*M* = 4.81, *SD* = 0.45), we selected six words per attribute which, according to the pre-testers, carry the meaning true or false, respectively. The mean agreement rating was *M* = 5.78 (*SD* = 1.03) for the six true-related words (e.g., *correct, proven*) and *M* = 5.14 (*SD* = 1.32) for the six false-related words (e.g., *wrong, faulty*). Four of the remaining words of the pretest were used as stimuli for the practice blocks. The complete list of the original German materials and their English translations is provided on the OSF.

### Design and procedure

The design was the same as in Experiment 2. Likewise, the procedure was similar to Experiment 2, except for the following changes. Participants had to categorize words displayed in red, green or gray as true-related or false-related. The practice phases preceding each test block comprised 12 trials (four words presented in three colors each). Each test block consisted of 72 trials in which the 12 target words (six true-related and six false-related words) appeared in each of the three colors twice (i.e., six times in total). The response deadline in block 2 was set to the 75th percentile of each participant’s RT distribution for correct responses in test block 1. We implemented this change based on test runs that had shown that stricter response deadlines (e.g., the 60th percentile as in the previous experiments) resulted in high proportions of missing responses. A final difference to Experiment 2 was that this time the experimenter administered the Ishihara test to identify participants with color blindness. Participants were shown six color plates in Ishihara’s (2003) test book.

## Results

### Ishihara color vision test

On average, participants identified *M* = 5.72 (*SD* = 0.68) of the six Ishihara plates correctly.^5^ Four participants were excluded from all subsequent analyses because they had difficulties correctly identifying the numbers displayed on at least two of the six color plates (see participant section).

### Perceptual fluency test

We analyzed mean RTs for correct responses of the perceptual fluency test (94% of the responses) by means of a 2 (target: present vs. absent) $\times$ 3 (color: green vs. red vs. gray) repeated-measures ANOVA. There was no color main effect, *F*(1.74, 73.18) = 1.02, *p* = 0.357, ${\eta }_{p}^{2}$ = 0.02, CI [0.00, 0.09], nor a target main effect or a color by target interaction, *F*s < 1. Hence, once again, the different colors were equally discriminable on the black screen.

### Stroop task

#### Response times

**Fig. 4:**
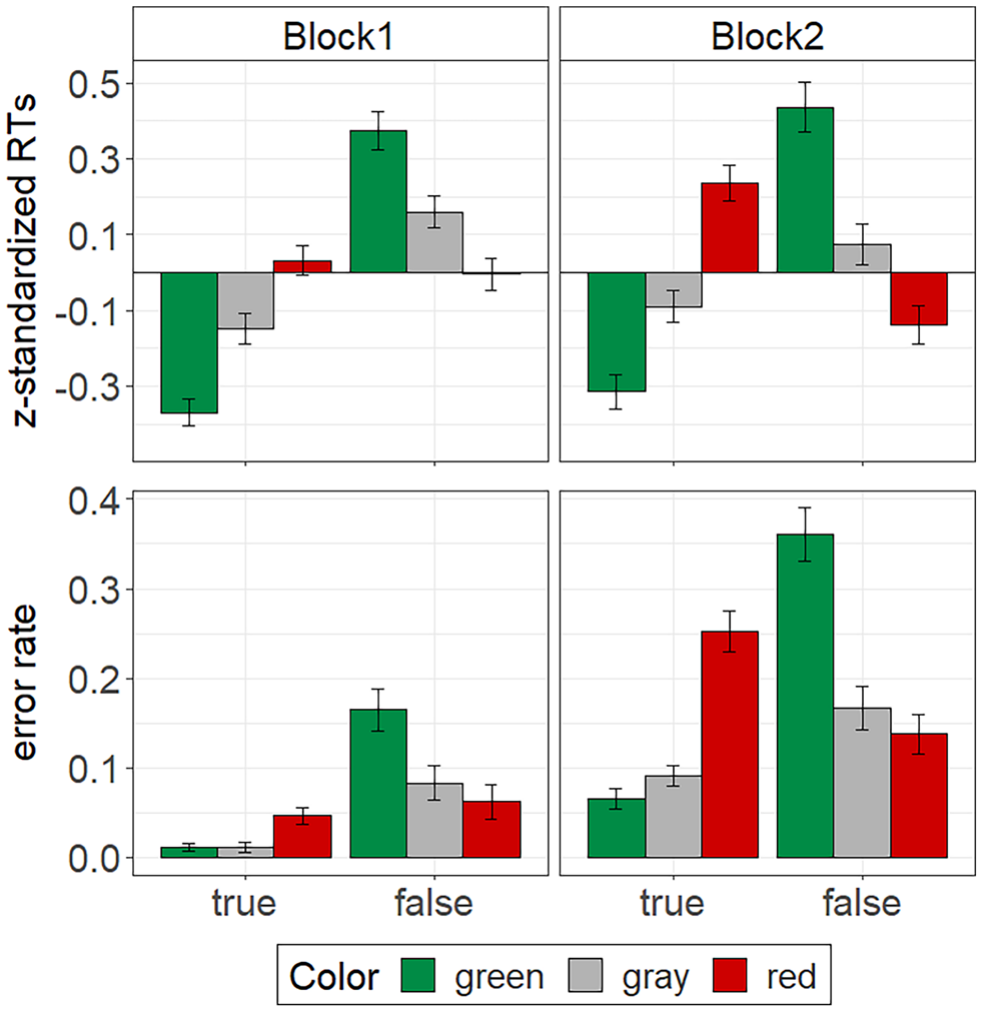
Stroop performance in Experiment 3 as measured by participants’ mean *z*-standardized RTs and participants’ error rates. The error bars represent standard errors of the means

In line with the prior experiments, we excluded RTs for incorrect responses (block 1: 6.3%, block 2: 14.6%) and omitted participants’ fastest and slowest correct response per block. We then *z*-standardized RTs for each participant per block to account for the different response deadlines of test block 1 (fixed deadline: 5000 ms) and test block 2 (adaptive response deadline: *M* = 814 ms, *SD* = 179 ms). The *z*-transformed RTs were submitted to a 2 (block: 1 vs. 2) $\times$ 3 (color: green vs. red vs. gray) $\times$ 2 (validity: true vs. false) repeated-measures ANOVA. Again, we report Greenhouse–Geisser corrected degrees of freedom whenever the sphericity assumption does not hold (as indicated by Mauchly’s test). All descriptive results are displayed in Fig. 4. Mean unstandardized RTs per condition are listed in Table 3.

**Table 3:** Mean (SD) unstandardized RTs and error rates for each condition of Experiment 3

Block	Validity	Color	RTs in ms	Errors in %
1	True	Green	665 (146)	1.2 (2.9)
		Gray	699 (124)	1.2 (3.4)
		Red	736 (158)	4.7 (6.4)
	False	Green	793 (155)	16.5 (15.5)
		Gray	768 (192)	8.3 (12.5)
		Red	736 (163)	6.2 (12.3)
2	True	Green	510 (56)	6.6 (7.6)
		Gray	528 (56)	9.1 (7.7)
		Red	553 (61)	25.2 (14.7)
	False	Green	570 (75)	36.0 (19.5)
		Gray	544 (67)	16.7 (15.5)
		Red	526 (56)	13.8 (14.3)

Similar to the previous experiments, participants responded faster to true-related words than to false-related words, *F*(1, 42) = 40.37, *p* < 0.001, ${\eta }_{p}^{2}$ = 0.49, CI [0.31, 0.62]. Once again, this validity effect was moderated by test block, *F*(1, 42) = 6.45, *p* = 0.015, ${\eta }_{p}^{2}$ = 0.13, CI [0.02, 0.30]. A comparison of simple main effects indicated a larger validity effect in test block 1, *F*(1, 42) = 60.44, *p* < 0.001, ${\eta }_{p}^{2}$ = 0.59, CI [0.43, 0.70], than in test block 2, *F*(1, 42) = 9.59, *p* = 0.003, ${\eta }_{p}^{2}$ = 0.19, CI [0.04, 0.36]. There was no significant color main effect on RTs, *F* < 1, but a significant color by validity interaction, *F*(1.93, 80.88) = 97.73, *p* < 0.001, ${\eta }_{p}^{2}$ = 0.70, CI [0.61, 0.76]. The latter was further qualified by a three-way interaction of block, color, and validity, *F*(1.95, 81.76) = 3.61, *p* = 0.032, ${\eta }_{p}^{2}$ = 0.08, CI [0.00, 0.17]. A separate analysis per test block showed that the color by validity interaction was considerably stronger in test block 2, *F*(1.85, 77.49) = 70.49, *p* < 0.001, ${\eta }_{p}^{2}$ = 0.63, CI [0.52, 0.70], than in test block 1, *F*(1.99, 83.44) = 35.84, *p* < 0.001, ${\eta }_{p}^{2}$ = 0.46, CI [0.33, 0.56]. However, importantly, the overall pattern was similar for both test blocks (see Fig. 4). The subsequent follow-up tests of the color by validity interaction therefore cover both blocks.

Simple main effect analyses indicated that color significantly affected RTs for true-related words, *F*(1.89, 79.36) = 61.19, *p* < 0.001, ${\eta }_{p}^{2}$ = 0.59, CI [0.48, 0.67], as well as false-related words, *F*(1.94, 81.57) = 40.64, *p* < 0.001, ${\eta }_{p}^{2}$= 0.49, CI [0.36, 0.59]. Planned comparisons within each validity condition with gray words as a the reference group yielded the following results: For true-related words, RTs were shorter when such words appeared in green, *F*(1, 42) = 34.30, *p* < 0.001, ${\eta }_{p}^{2}$= 0.45, CI [0.26, 0.59], and longer when they appeared in red, *F*(1, 42) = 35.17, *p* < 0.001, ${\eta }_{p}^{2}$= 0.46, CI [0.27, 0.60]. Vice versa, for false-related words, RTs were shorter when such words appeared in red, *F*(1, 42) = 16.57, *p* < 0.001, ${\eta }_{p}^{2}$= 0.28, CI [0.11, 0.45], and longer when they appeared in green, *F*(1, 42) = 24.71, *p* < 0.001, ${\eta }_{p}^{2}$= 0.37, CI [0.18, 0.53].

#### Accuracy

We compared mean error rates for the different combinations of block, color, and validity by means of a 2 $\times$ 3 $\times$ 2 repeated-measures ANOVA. The descriptive results are illustrated in Fig. 4. As in the previous experiments, participants made more errors in test block 2 than in test block 1, *F*(1, 42) = 130.20, *p* < 0.001, ${\eta }_{p}^{2}$ = 0.76, CI [0.65, 0.82]. Moreover, the error rate was higher for false-related words than for true-related words, *F*(1, 42) = 22.00, *p* < 0.001, ${\eta }_{p}^{2}$ = 0.34, CI [0.16, 0.50]. Additionally, there was a main effect of color, *F*(1.98, 83.20) = 28.99, *p* < 0.001, ${\eta }_{p}^{2}$ = 0.41, CI [0.27, 0.52]. The latter effect was qualified by a color by block interaction, *F*(1.93, 80.91) = 6.04, *p* = 0.004, ${\eta }_{p}^{2}$ = 0.13, CI [0.03, 0.23], a color by validity interaction, *F*(1.37, 57.50) = 72.39, *p* < 0.001, ${\eta }_{p}^{2}$ = 0.63, CI [0.53, 0.71], and a significant three-way interaction of block, color, and validity, *F*(1.76, 73.85) = 30.74, *p* < 0.001, ${\eta }_{p}^{2}$ = 0.42, CI [0.29, 0.53]. Similar to the RT data, the color by validity interaction was considerably stronger in test block 2, *F*(1.51, 63.25) = 68.18, *p* < 0.001, ${\eta }_{p}^{2}$ = 0.62, CI [0.51, 0.69], than in test block 1, *F*(1.62, 67.87) = 23.62, *p* < 0.001, ${\eta }_{p}^{2}$ = 0.36, CI [0.22, 0.47]. Importantly, however, the overall pattern was similar for both test blocks (see Fig. 4, for exact values of mean percent errors per condition see Table 2). Thus, again, the subsequent follow-up tests of the color by validity interaction cover both blocks.

Simple main effect analyses indicated that color significantly affected response accuracy for true-related words, *F*(1.49, 62.67) = 54.00, *p* < 0.001, ${\eta }_{p}^{2}$ = 0.56, CI [0.44, 0.65], as well as for false-related words, *F*(1.51, 63.37) = 59.24, *p* < 0.001, ${\eta }_{p}^{2}$ = 0.59, CI [0.47, 0.67]. Planned comparisons within each validity condition revealed that participants’ mean error rates were significantly higher for the true-related words displayed in red than for such words displayed in gray, *F*(1, 42) = 60.84, *p* < 0.001, ${\eta }_{p}^{2}$= 0.59, CI [0.43, 0.70]. In contrast, mean error rates did not significantly differ between true-related words displayed in green and gray, *F*(1, 42) = 2.70, *p* = 0.108, ${\eta }_{p}^{2}$= 0.06, CI [0.00, 0.21]. The opposite pattern emerged for the false-related words. Mean error rates were considerably higher for false-related words presented in green than in gray, *F*(1, 42) = 67.20, *p* < 0.001, ${\eta }_{p}^{2}$= 0.62, CI [0.46, 0.72], and somewhat lower when such words were presented in red compared to gray, *F*(1, 42) = 5.23, *p* = 0.027, ${\eta }_{p}^{2}$= 0.11, CI [0.01, 0.27].

### Explicit color–validity associations

When asked about their explicit color–validity associations, all participants indicated that they associated green with the attribute true and red with the attribute false. A complete list of participants’ explicit color associations is provided in Appendix A.

## Discussion

The results of Experiment 3 provide clear evidence for the hypothesized green–true and red–false associations. This time, the associations showed up in participants’ RTs as well as in their error rates and were evident in both test blocks. Similar to the previous experiments, however, the associations were more pronounced in test block 2 than in test block 1 as indicated by follow-up tests on the three-way interactions of block, color, and validity. Effects of green–true and red–false associations on Stroop performance thus appear to be particularly strong under speeded conditions.

## General discussion

The goal of this research was to systematically investigate automatic color–validity associations with a Stroop-like paradigm. In a series of three experiments, we found clear evidence that people associate the colors green and red with the attributes true and false, respectively. More precisely, participants’ performance in the Stroop task (as measured by participants’ RTs and accuracy) varied as a function of stimulus color and validity. In Experiment 1, however, this color by validity interaction only emerged when green and red statements appeared within the same context. For the accuracy data in the green–red condition, this interaction was further moderated by test block. Follow-up analyses showed that the predicted color by validity interaction only appeared in test block 2 in which participants had to provide speeded responses. Similarly, in Experiment 2, the Stroop effect for the RT data solely replicated under speeded conditions. For the accuracy data, the effect did not replicate at all. Finally, in Experiment 3, which used simpler materials (i.e., words instead of statements), we found a strong color–validity interaction for the RT data as well as the accuracy data regardless of whether participants had to respond particularly fast or not. However, in line with the findings of Experiments 1 and 2, the effects were significantly stronger under speeded conditions.

In order to test whether our results are robust when choosing a different methodological approach, we also analyzed the Stroop data of all three experiments with (generalized) linear mixed-effects models (see Appendix B). The results were largely consistent with the ANOVA findings. The most apparent difference was that the three-way interaction of block, color, and validity did not replicate in two cases when analyzing the accuracy data with generalized linear mixed-effects models. Note, however, that the test of this three-way interaction was possibly underpowered as our a-priori power-analyses had been targeted to detect a two-way interaction of color and validity with a repeated-measures ANOVA. Most importantly, the results of the linear mixed-effects model analyses do not challenge the following conclusions that we draw based on our ANOVA findings.

In line with the prediction of the dimension-specificity hypothesis, green–true associations and red–false associations are moderated by color contexts. Similar to other color-meaning associations these associations get only activated if a color with opposite meaning occurs in the same context (e.g., Schietecat et al. 2018b). This becomes particularly evident in Experiment 1, where we found color–validity associations in the green–red condition, but not in the green–gray or the red–gray condition. Moreover, our findings suggest that controlled processing can override the influence of automatic color associations on Stroop performance. This conclusion rests on the following observations: First, in test block 1, which allowed more controlled responding than test block 2, color–validity interactions tended to be less strong. In some cases, color–validity effects were even completely absent in test block 1. Second, in Experiments 1 and 2 we found the predicted color effects for true statements only. As noted previously, the processing of false statements presumably involves more elaborate processing than the processing of true statements, which might have counteracted the influence of automatic color effects on such statements. Finally, the finding that color–validity interactions were generally stronger in Experiment 3 also seems to relate to the fact that more cognitive processing is needed to evaluate the validity of statements than the meaning of individual words.

One point that needs further consideration is the interpretation of the observed color–validity interactions in terms of facilitation versus interference effects. Given the lack of a reference category in Experiment 1, we were unable to determine whether the faster RTs and higher accuracy for the true statements in green versus red represented a facilitation effect of the association-congruent color (green) or an interference effect of the association-incongruent color (red). When adding gray as reference color in Experiment 2, we found no significant facilitation effect on RTs for true statements displayed in green, albeit the descriptive results were in the expected direction. Likewise, there was no significant interference effect on RTs for true statements displayed in red. But, again, the descriptive results were in the expected direction. Finally, in Experiment 3 stimulus color not only affected RTs and the accuracy of “true” responses, but also of “false” responses. Moreover, this time, we observed both, interference effects of association incongruent colors (red–true; green–false) and facilitation effects of association congruent colors (green–true; red–false). However, the latter effects were less strong and were only observed under speeded conditions (i.e., in block 2). Finally, the fact that green-effects and red-effects were quite symmetrical (albeit in opposite directions), suggests that automatic green-true associations and automatic red-false associations are about equally strong.

Symmetric color–validity associations were also found at the explicit level. In Experiments 2 and 3, for example, the proportion of participants indicating an explicit green–true association was exactly the same as the proportion of participants indicating an explicit red–false association. In Experiment 1, in contrast, green–true associations were reported less often than red-false associations. However, it should be noted that the questionnaires used to assess explicit associations were not identical across experiments. The questionnaire in Experiment 1 asked participants to indicate their color associations for “truth” and “falsity” whereas the questionnaires in Experiments 2 and 3 asked participants to indicate their color-associations for the attributes “true” and “false”. This difference in terminology may have had an impact on participants’ explicit color associations. Overall, the findings suggest that green and red are more strongly associated with the attributes true and false than with the concepts “truth” and “falsity”. Interestingly, Meier et al. (2004) stated that “In Buddhist writings, truth is characterized as a light or a lamp, with seekers of truth shining brightly.” (p. 82). Similarly, a considerable number of participants in Experiment 1 indicated to associate truth with the color white. However, importantly, this number was below the number of reported green–truth associations.

Because all of our results rest on samples of German University students only, research is needed to investigate whether people with different cultural backgrounds share the same color–validity associations. Cultural differences would suggest that such associations are socially learned. Building on this, it would be interesting to explore at what age color-validity associations start to form and which contexts and experiences lead to such associations. A recent study by Hong et al. (2020) with Chinese participants, for example, found green–success and red–failure associations among college students, but reverse associations among a group of stock shareholders, and no such associations among a more general sample of adults who did not attend university and had no experiences in stock trading. This finding suggests that different social learning experiences might lead to different color–validity associations (see also Jiang et al. 2014).

Besides the need for more diverse samples, future research should also test the robustness of our findings by using different materials, tasks, and experimental procedures. For instance, although we could show that colors may affect the speed and accuracy of true/false responses, it remains unclear whether the validity of statements conversely affects color identification. Moreover, other implicit measures, such as the IAT (e.g., Schietecat et al. 2018b) or the truth misattribution procedure (TMP, Cummins & Houwer, 2019), could be utilized to explore the generalizability of our findings. Finally, future studies might investigate the impact of color–validity associations on more complex psychological functions such as judgments and decision-making processes as well as memory processes. For instance, people might be more inclined to judge a statement of unknown validity to be true if the statement is printed in green than if it is printed in black or red. Likewise, color–validity associations could improve or impair memory for the validity of information. For instance, displaying true information in green and false information in red could help people to memorize truth and falsity, respectively (see Pantazi et al. 2018).

## Conclusion

To our knowledge, our study is the first to investigate color–validity associations. In three experiments, we tested whether people hold green–true and red–false associations by investigating color effects on the speed and accuracy of true/false responses. In sum, our experiments provide empirical support for the hypothesized green–true and red–false associations. However, the results also show that these color–validity associations depend both on the color context and on the extent to which controlled processes are involved in processing the task at hand. We are convinced that these findings provide a fruitful basis for further research on the impact of color–validity associations on psychological functioning.

## Data Availability

The materials and all datasets generated and analyzed for this article are publicly available online at the Open Science Framework (OSF; https://osf.io/b8wux/).

## Appendix A

Table 4 provides a complete list of participants' explicit color associations for all experiments.

**Table 4:** Explicit color-validity associations

Color	Experiment 1		Experiment 2		Experiment 3	
	True	False	True	False	True	False
Green	45	1	40	0	43	0
Red	1	58	0	40	0	43
White	29	0	6	0	13	0
Black	4	12	1	6	2	9
Gray	1	4	0	0	0	0
Yellow	3	5	0	1	0	1
Blue	10	1	2	0	3	0
Pink	1	0	0	1	0	0
Purple	1	2	0	0	0	2
Brown	0	1	0	0	0	0
Orange	0	7	0	0	0	10
None	15	12	0	0	0	0

*N*_*Exp1*_ = 75, *N*_*Exp2*_ = 43, *N*_*Exp3*_ = 43. Naming/choosing multiple colors was possible

The questions on explicit color–validity associations differed between the experiments in terms of wording and response format. In Experiment 1, we asked participants if they associated one or several colors with the concepts “truth” and “falsity”. Response options for each concept were “No” and “Yes, that is …”, the latter being followed by multiple choice options (blue, green, yellow, red, orange, purple, black, white, gray, others such as…). In Experiment 2, we asked participants if they associated one or several colors with the attributes “true” and “false”. Response options were “No” and “Yes, that is…” with a blank field where participants could write down their answer. Experiment 3 also asked for color-associations with the attributes “true” and “false”, but the response format was changed back to multiple choice.

## Appendix B

In order to assess the robustness of our findings when using a different methodological approach, we additionally analyzed the Stroop data of all experiments with (generalized) linear mixed-effects models. We ran the analyses with the statistics software R (version 3.6.2) with the function *mixed* from the package *afex* (Singmann et al. 2020) which uses the packages *lme4* (Bates et al. 2015) and *lmerTest* (Kuznetsova et al. 2017) for model estimation. We analyzed the RT data with linear mixed-effects models based on restricted maximum likelihood estimation (REML). *P*-values were calculated using Satterthwaite approximation for degrees of freedom. For the accuracy data, we fitted generalized linear mixed-effects models with binomial family and logit link function using maximum likelihood estimation (Laplace approximation). *P*-values were calculated using likelihood-ratio tests. All models included the contrast-coded fixed factors block, color, and validity, and all possible interactions between these factors. Regarding the random effects, the data neither allowed to estimate a maximal random effects model (Barr et al. 2013) nor a model including random slopes for interactions of interest (Barr 2013), as indicated by singular-fit warnings (Singmann and Kellen 2019). For this reason, we only fitted random-intercept models. Although these models may have a higher Type-I error rate than ANOVA (Barr 2013), their Type-I error rate is likely lower than that of models that include random slopes for main effects, but lack random slopes for interactions of interest (see Barr 2013). For the *z*-standardized RT data, our models included random intercepts for items, but not for participants. The latter did not differ in their intercept due to the *z*-standardization. For the accuracy data, the models included random intercepts for items and participants. The results of all models compared to the ANOVA findings are displayed in Tables 5, 6, 7.

**Table 5:** Results of the linear mixed model analyses (LMM) and generalized linear mixed model analyses (GLMM) compared to the ANOVA results for each color-context condition of Experiment 1

Results per condition		RT data		Accuracy data	
		LMM	ANOVA	GLMM	ANOVA
Green–gray condition					
	Block	*F* < 1	*F* = 1	χ^2^ = 244.40***	*F* = 90.34***
	Color	*F* = 2.00	*F* = 1.78	χ^2^ < 1	*F* < 1
	Validity	*F* = 29.69***	*F* = 76.22***	χ^2^ = 3.00 +	*F* < 1
	Block $\times$ Color	*F* < 1	*F* < 1	χ^2^ < 1	*F* < 1
	Block $\times$ Validity	*F* = 33.75***	*F* = 28.57***	χ^2^ = 11.42***	*F* = 7.26*
	Color $\times$ Validity	*F* < 1	*F* < 1	χ^2^ = 1.21	*F* < 1
	Block $\times$ Color $\times$ Validity	*F* < 1	*F* < 1	χ^2^ = 1.75	*F* < 1
Red-gray condition					
	Block	*F* < 1	*F* < 1	χ^2^ = 172.54***	*F* = 91.73***
	Color	*F* < 1	*F* < 1	χ^2^ < 1	*F* < 1
	Validity	*F* = 19.44***	*F* = 35.83***	χ^2^ = 3.15 +	*F* < 1
	Block $\times$ Color	*F* < 1	*F* < 1	χ^2^ < 1	*F* < 1
	Block $\times$ Validity	*F* = 18.35***	*F* = 17.87***	χ^2^ = 7.53**	*F* = 4.13 +
	Color $\times$ Validity	*F* < 1	*F* < 1	χ^2^ = 2.63	*F* = 2.93 +
	Block $\times$ Color $\times$ Validity	*F* < 1	*F* < 1	χ^2^ < 1	*F* < 1
Green–red condition					
	Block	*F* < 1	*F* = 1.15	χ^2^ = 262.77***	*F* = 57.32***
	Color	*F* = 1.57	*F* = 2.50	χ^2^ < 1	*F* = 1.25
	Validity	*F* = 13.39***	*F* = 45.53***	χ^2^ = 5.51*	*F* < 1
	Block $\times$ Color	*F* < 1	*F* < 1	χ^2^ < 1	*F* = 1.28
	Block $\times$ Validity	*F* = 6.89**	*F* = 6.04***	χ^2^ = 13.55***	*F* = 4.23*
	Color $\times$ Validity	*F* = 9.64**	*F* = 9.36**	χ^2^ < 1	*F* = 1.69
	Block $\times$ Color $\times$ Validity	*F* < 1	*F* < 1	χ^2^ = 2.70	*F* = 5.53*

****p* < .001, ***p* < .01, **p* < .05, + *p* < .10

**Table 6:** Results of the linear mixed model analysis (LMM) and the generalized linear mixed model analysis (GLMM) compared to the ANOVA results of Experiment 2

	RT data		Accuracy data	
	LMM	ANOVA	GLMM	ANOVA
Block	*F* < 1	*F* = 1.69	χ^2^ = 369.16***	*F* = 144.36***
Color	*F* = 3.82*	*F* = 3.56*	χ^2^ < 1	*F* < 1
Validity	*F* = 20.11***	*F* = 98.22***	χ^2^ < 1	*F* < 1
Block $\times$ Color	*F* < 1	*F* < 1	χ^2^ < 1	*F* < 1
Block $\times$ Validity	*F* = 17.28***	*F* = 20.36***	χ^2^ = 3.41 +	*F* = 1.71
Color $\times$ Validity	*F* = 3.27*	*F* = 3.05 +	χ^2^ = 1.58	*F* = 1.03
Block $\times$ Color $\times$ Validity	*F* = 3.65*	*F* = 4.09*	χ^2^ < 1	*F* < 1

^***^*p* < .001, ***p* < .01, **p* < .05, + *p* < .10

**Table 7:** Results of the linear mixed model analysis (LMM) and the generalized linear mixed model analysis (GLMM) compared to the ANOVA results of Experiment 3

	RT data		Accuracy data	
	LMM	ANOVA	GLMM	ANOVA
Block	*F* = 1.93	*F* = 11.37**	χ^2^ = 215.28***	*F* = 130.20***
Color	*F* < 1	*F* < 1	χ^2^ = 15.95***	*F* = 28.99***
Validity	*F* = 3.83 +	*F* = 40.37***	χ^2^ = 4.43*	*F* = 22.00***
Block $\times$ Color	*F* < 1	*F* < 1	χ^2^ < 1	*F* = 6.04**
Block $\times$ Validity	*F* = 9.93**	*F* = 6.45*	χ^2^ = 15.31***	*F* < 1
Color $\times$ Validity	*F* = 116.41***	*F* = 97.73***	χ^2^ = 132.78***	*F* = 72.39***
Block $\times$ Color $\times$ Validity	*F* = 3.51*	*F* = 3.61*	χ^2^ = 1.35	*F* = 30.74***

****p* < .001, ***p* < .01, **p* < .05, + *p* < .10

## Footnotes

1 We would like to thank Edgar Erdfelder, one of the G*Power authors, for his advice and support in conducting the power-analyses with the Generic *F*-test module.

2 Our final sample sizes were somewhat smaller than our target sample sizes. However, even in the condition with the smallest number of participants (green-gray condition), the estimated power to detect the expected color $\times$ validity interaction was still high (1-β = .90).

3 One participant whose level of German was not good enough was excluded before analyzing the Ishihara test.

4 Confidence intervals (CIs) for the effect size measure ${\eta }_{p}^{2}$ were computed with the R-package *effectsize* (Ben-Shachar et al., 2020). These CIs always denote 90% CIs (see Lakens, 2014). In cases in which multiple CIs are reported, the first refers to the green-gray condition, the second to the red-gray condition, and the third to the green–red condition.

5 One participant with language difficulties was excluded before analyzing the Ishihara test.

## Code availability

The R code used to analyze the data is available upon request.
